# Children and Older Adults Exhibit Distinct Sub-Optimal Cost-Benefit Functions when Preparing to Move Their Eyes and Hands

**DOI:** 10.1371/journal.pone.0117783

**Published:** 2015-02-06

**Authors:** Claudia C. Gonzalez, Mark Mon-Williams, Melanie R. Burke

**Affiliations:** 1 Sports, Health and Exercise Sciences, Brunel University, Uxbridge, United Kingdom; 2 School of Psychology, Faculty of Medicine and Health, University of Leeds, Leeds, United Kingdom; University of Leicester, UNITED KINGDOM

## Abstract

Numerous activities require an individual to respond quickly to the correct stimulus. The provision of advance information allows response priming but heightened responses can cause errors (responding too early or reacting to the wrong stimulus). Thus, a balance is required between the online cognitive mechanisms (inhibitory and anticipatory) used to prepare and execute a motor response at the appropriate time. We investigated the use of advance information in 71 participants across four different age groups: (i) children, (ii) young adults, (iii) middle-aged adults, and (iv) older adults. We implemented ‘cued’ and ‘non-cued’ conditions to assess age-related changes in saccadic and touch responses to targets in three movement conditions: (a) Eyes only; (b) Hands only; (c) Eyes and Hand. Children made less saccade errors compared to young adults, but they also exhibited longer response times in cued versus non-cued conditions. In contrast, older adults showed faster responses in cued conditions but exhibited more errors. The results indicate that young adults (18–25 years) achieve an optimal balance between anticipation and execution. In contrast, children show benefits (few errors) and costs (slow responses) of good inhibition when preparing a motor response based on advance information; whilst older adults show the benefits and costs associated with a prospective response strategy (i.e., good anticipation).

## Introduction

The neurophysiological limits of information processing produce temporal lags in a human’s response to environmental change. The existence of response delays is potentially detrimental from an evolutionary perspective so it is unsurprising that humans have developed neural mechanisms that can exploit information to prepare a motor response in advance of an anticipated change in the environment. These mechanisms have been investigated by researchers using ‘cueing’ techniques where advance information (e.g., a target’s location) is provided prior to the presentation of a stimulus [1]. Thus, for example, cues have been shown to facilitate the generation of a saccadic eye movement [2,3].

There are clear advantages to using advance information to prepare a response. Nevertheless, there are also potential costs if the response is generated too early or triggered by an irrelevant stimulus. It follows that skilled behaviour requires a balance between priming a potentially required response (anticipation) and inhibiting the prepared movement prior to the appearance of the appropriate stimulus. This inhibition-anticipation balance is advantageous in a number of activities in which preparing a response is essential but responding before the appropriate time is detrimental (e.g., in tennis, goalkeeping and while driving). Thus, the *advantage* resulting from the early programming of a motor response is highly dependent on online cognitive processes that include the ability to inhibit reactive responses to allow for the volitional response to be executed [4]. For example, a saccadic eye movement during a task that requires the active process of maintaining fixation indicates inappropriate allocation of attention, which may result in accuracy *costs* with poor acquisition of information [5], and/or latency *costs* (e.g., inhibition of return, [6]).

Mon-Williams and colleagues [7] [8] have shown that even young children (4–5 years) can utilise cue information to decrease their reaction times in a manual aiming task. Similarly, older adults have also been found to use cue information to plan an upcoming response [9], but these responses have been found to be slower compared to younger adults and often show speed-accuracy trade-offs [10–12]. Sweeney et al. [5] found that older participants had difficulty in inhibiting eye movements towards flashed targets and exhibited less accurate saccades to a cued target than younger adults. Fischer et al. [13] reported that anti-saccade errors to cues are present in children but these errors decrease with increasing age, with a moderate deterioration in performance observed in older participants (>40 years of age) (also see [14,15]). Furthermore, a number of neurophysiological and behavioural studies suggest that older adults implement ‘strategies’ to compensate for detrimental age-related changes in motor control (for review see [16]).

The ballistic nature of saccades allows for the ideal investigation of such inhibition/anticipation mechanisms and indeed studies have reported that volitional control of saccades is often influenced by cognitive factors such as attention, inhibition, decision-making and working memory for planning, which have all been shown to be impaired with ageing [13] and show greater variability in children [13,14,17]. Hand movements are not ballistic with their slower movement time allowing for on-line feedback during the response. The primary aim of the study, however, was not to address differences between these systems, but to assess the contributions of each to produce accurate and timely responses in the differing age groups, as this has not yet been fully explored. Thus, we investigated the age-related effects of the cost-benefit function by measuring saccade and touch responses in four different age groups (children, young adults, middle aged adults and older adults). This approach aimed to provide metrics of inhibition and anticipation via accuracy and timing measures and help establish how cost-benefit balances change from children to older adults. We hypothesised that learning the optimum cost-benefit functions for specific tasks would mature over childhood. We anticipated that children would show increased errors (incorrect responses) when presented with cue information compared to the adult group. Our prediction was based on the conjecture that the children would have had insufficient developmental experience to adopt an optimum cost-benefit function. There is empirical evidence to suggest that children make more errors when they adopt predictive strategies. For example, it has been reported that children exhibit a decreased ability to supress reactive saccades during fixation tasks [17] and show higher error rates during anti-saccade tasks [13,14,18]. In general it has been found that incorrect responses decrease with age over childhood [19], although the exact age of maturation of oculomotor cognitive control tends to vary between studies and tasks (for review see [20]). Observed developmental limitations in visual fixation suggest that these mechanisms are related to higher order online cognitive control processes [20].

Similarly, reports of age-related declines in online cognitive processing have been suggested to occur [5,21]. We therefore also hypothesised that older adults would show sub-optimal cost-benefit functions in an eye-hand coordination task. We anticipated that older adults would either show increased errors (incorrect responses) and/or differences in movement times when presented with cue information (though we could not predict whether the differences would be in movement time or error rate). The prediction of differences in performance was based on known declines in the control of motor responses such as saccadic initiation [5,13]. In addition, older adults have been shown to exhibit longer movement times than younger adults, particularly in the deceleration phase (feedback control) during planned visually-guided aiming responses [11,22]. We therefore explored how these inhibitory deficiencies would affect eye and hand responses to cued targets.

These predictions were tested in the four age groups using three tasks: i) eyes only, ii) hands only (while maintaining central fixation), and iii) eyes and hand. The effects of cues in eye movements [3] and hand movements [1] have been previously described. Our aim was to compare between the three tasks and provide insight into the interactions between the eye and the hand and how inhibitory and anticipatory mechanisms are integrated during coordinated actions across the different age-groups. We expected that concurrent hand movements would be affected (i.e., accuracy and/or timing costs) if children and older adults did show more inhibitory errors in saccadic eye movements. It was not known, however, whether the number of errors and resulting performance costs would differ across the three conditions, since existing studies typically report results in terms of hand reaction times and eye movements are not measured.

## Methods

### Participants

Seventy one participants between the ages of 8 and 79 years (yrs) were recruited and divided into 4 age groups, based on previous research investigating changes in the maturation and age-related effects of saccade generation and inhibition (see [5,18]): 1) Children (CH, 8–12 yrs, n = 16, mean age = 9.9 ± 1.5 yrs, 4 females and 12 males); 2) Young adults (YA, 21–25 yrs, n = 20, mean age = 21.3 ± 0.98 yrs, 14 females and 6 males); 3) Middle-aged adults (MA, 30–45 yrs, n = 16, mean age = 37.4 ± 6.3 yrs, 9 females and 7 males); and 4) Older adults (OA, 60–80 yrs, n = 19, mean age = 65.2 ± 5.9 yrs, 12 females and 7 males). All participants were determined to have normal or corrected eyesight and provided details of prescriptions if glasses or contact lenses were worn. We additionally assured visual acuity in all subjects by using a Snellen scale on the day of testing with all subjects achieving 6/6 m. All subjects reported no known neurological or developmental conditions.

### Ethics statement

All participants gave informed written consent and they were informed that they could stop the experiment at any point prior to the experimental sessions. In the case of children (<18 yrs), additional informed written consent was also provided from their parent or guardian. This study and consent procedure were approved by the University of Leeds ethics committee and conducted in accordance with the ethical standards laid out in the 1964 Declaration of Helsinki and the British Psychology Society (BPS) guidelines.

### Experimental setup

Participants were seated on an adjustable chair with their heads supported by a chin and forehead rest, to restrict head movements, 38 cm from a touch screen computer (19 inch colour CRT monitor, 1024 by 768 pixel resolution, with a refresh rate of 75Hz, touch screen activation force of 50–120 grams per square centimetre and an accuracy that exceeds 0.3 cm, Elo Touch Solutions, Inc.). Stimuli were presented using Experiment Builder software (SR research, Canada), while eye movements were recorded using an eye-tracker sampling at 1000 Hz (Eyelink 1000, SR research, Canada). A separate computer recorded and stored the data for subsequent offline analysis. All visual stimuli were 1.3 x 1.3 degrees of visual angle (50 pixels in diameter) and presented on a black background (luminance of 50 cd/m^2^). Experimental sessions took place in a dark quiet room to avoid any distractions. Rests were provided between each experimental block and when needed. The lights were turned on during these rest periods to maintain alertness and minimize dark adaptations. Experimental sessions lasted for less than 60 min.

### Experimental protocol

**Fig 1:**
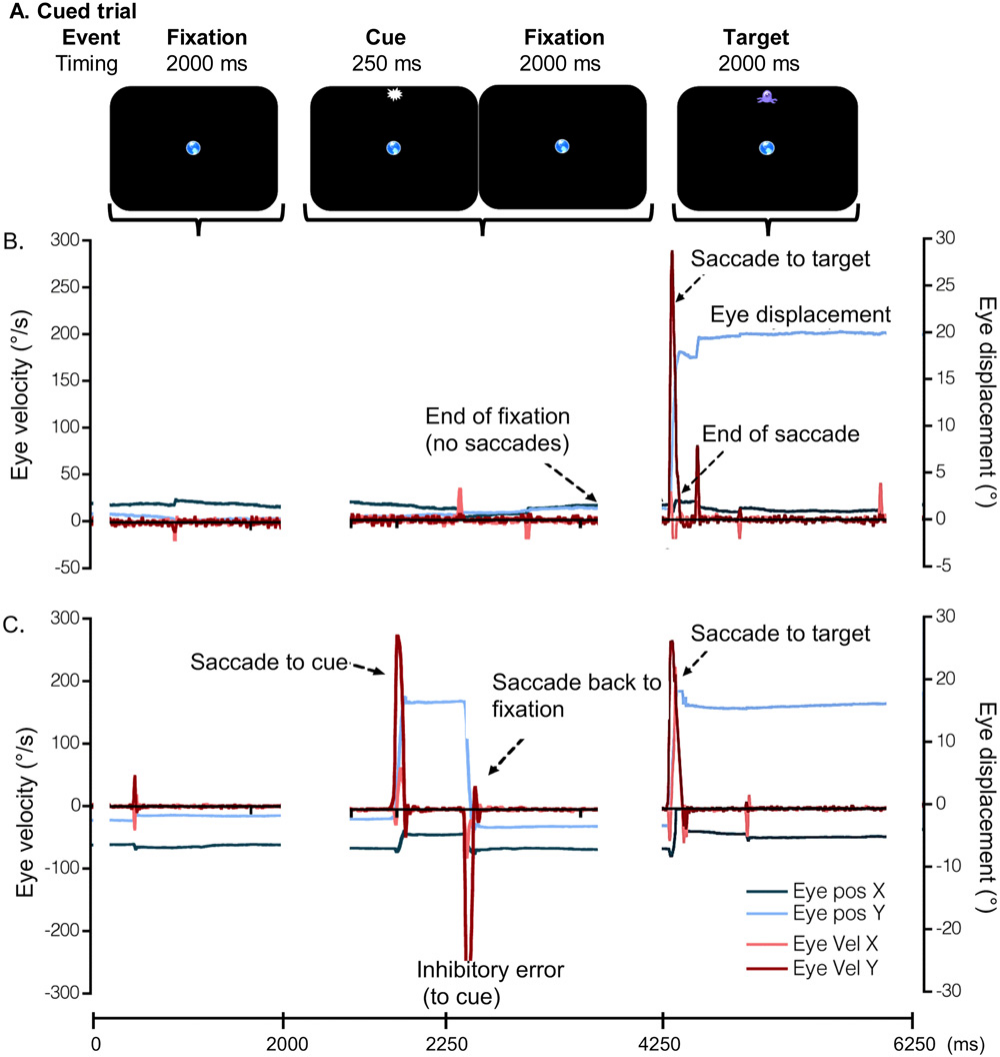
Cued trial events and durations (A) and examples of Cued trials from a young (B) and an older adult (C) participants. Targets (purple alien images) could appear at one of four locations at 9° from the centre fixation (blue world image). The graphs show eye displacement (Eye pos) and velocity (Eye Vel) in X and Y across the Cued trial events (i.e., Fixation, Cue, and Target) (A) and across time (X axis). Graph B shows one saccade made in response to the target, whilst graph C shows one saccade in response to the cue (i.e., inhibition error), a second saccade back to the centre fixation when the participant became aware of the error, and a third saccade in response to the target. The central fixation was visible throughout the experimental trials. Engaging targets were used to encourage children to perform the experiment and these appear larger for schematic purposes.

For the **Cued (C)** task, a central fixation point was presented for 2000 ms, after which a cue (prior to target) was presented 9° from the centre in one of 4 locations along the horizontal and vertical axis (at 90°, 180°, 270° and 360°) for 250 ms. A target then appeared 2000 ms after the cue offset, in the same location as the cue and remained visible for 2000 ms for subjects to make their response (see Fig. 1A). The cue was always valid and all participants were asked to inhibit any type of response to the cue and maintain fixation on the centre of the screen until the target appeared. For the **Non-Cued (NC)** task, participants fixated a central target (0°) for 1500 or 2500ms, after which a target appeared in one of the 4 locations mentioned above. This target remained visible either 1500 or 2500ms. Both fixation and target timings (1500 or 2500 ms) were randomized and balanced between trials within each experimental NC block. The central fixation point was present throughout the C and NC trials, but disappeared with the target to signal the start of a new trial (inter-trial time of 1000 ms). Target and cue locations were counter-balanced between experimental blocks and participants.

All participants were asked to perform the C and NC tasks within three conditions: 1) eye only (EO); 2) eye and hand (EH); and 3) hand only (HO). For the **EO** trials, participants were asked to fixate at the central fixation point and then fixate at the target when appearing at one of the four target locations. For the **EH** trials, participants were instructed to look and touch the fixation point at the beginning of each trial and then look at and touch the target with their dominant (preferred) hand on the touch screen computer using their index finger (as accurately and quickly as possible). The **HO** block trials consisted of responding to the target using only their hand while maintaining fixation upon the central fixation point at all times.

There were a total of 6 experimental blocks (C and NC tasks x 3 conditions: EO, HO and EH) and each consisted of 32 trials. Participants were asked to respond to the target as fast and as accurately as possible and they were aware that their reaction time and accuracy to the target would be measured throughout the experimental session, but no feedback was provided on their performance. Engaging stimuli (pictures of the earth, a blast and an alien as fixation, cue and target respectively) were used to motivate the children to perform the experiments. The same stimuli were used and the same instructions were given to all participants of all age groups. The 2 cued and non-cued conditions and 3 eye and/or hand tasks (6 conditions in total) were randomized between the adult participants, but blocked in the following order for the children in order to avoid confusion between tasks: Non-Cued EO, EH and HO; and Cued EO, EH and HO (also see[23]). Each experimental block started with a 5 point calibration, followed by a validation of the eye position based on this initial calibration. Practice trials were introduced at the beginning of each block under close observation of the experimenter to ensure that all participants were performing the tasks correctly and to make sure that the participant got used to the task, eliminating practice-related effects.

### Data analysis

Participants’ touch and eye movement data were obtained from the Data Viewer software (SR research, Canada). Blinks were automatically eliminated from the raw data before analysis and the gaps corresponding to the eliminated blinks were bridged using linear interpolation methods. Eye displacements and velocities were analysed using a custom made programme in MatLab (version 13a, The Mathworks, Inc.) for saccade identification and quantification. Saccades were computed from the horizontal and vertical velocity traces and identified as samples with an instantaneous velocity exceeding 100°/s (Fig. 1B and 1C). Saccade onsets were obtained using differentiation techniques (peak jerk) [24] and latencies were computed from target onset to saccade onset. Saccades made in response to the cue were measured separately as inhibition errors and eliminated from the latency eye data analyses (for an example of an error trial see Fig. 1C). Given the predictive nature of the Cued task (known locations and predictable timings), it was expected that participants would exhibit anticipatory responses. Overall, anticipatory saccades (latency < 80 ms) amounted to 9 ± 11.9%, 7.1 ± 9.1%, 17.23 ± 20.1% and 14.36 ± 15.04% of EO trials and 9.7 ± 11.4%, 12.69 ± 14.6%, 18.47 ± 15.03% and 36.82 ± 30.7% of EH trials for CH, YA, MA and OA respectively. Thus, anticipatory saccades were included in the analysis and defined as predictive responses with latencies between-500 and 80 ms (i.e., prior to obtaining visual feedback of the target but also not as a response to the cue) [25]. However, anticipatory saccades were eliminated from the NC tasks as these would correspond to guesses and these only occurred in < 2% of trials in each block for the young, middle-aged and older adult group and no anticipatory responses or guesses were observed in Children. Eye accuracy was measured in terms of absolute error (the magnitude or distance of the eye response from the target or fixation point irrespective of direction), constant error (the directional error from the target) and variable error (the standard deviation of responses). Eye displacements were obtained by subtracting the end position of the eye following the saccade (averaged over 500 ms) during the initial fixation and target.

Touch time was defined as the time from the start of the target onset until the participant touched the target. Touch accuracy was also measured in terms of absolute error, constant error, and variable error from the target. Trials in which participants made eye movements to the cue in Cued trials or to the target during Cued and Non-Cued HO trials were also eliminated from the touch analysis (see above).

Eye and hand data were fed into a multivariate design using a mixed measure analysis of variance (ANOVA) (SPSS version 20, IBM, USA). Group differences were evaluated using a Bonferroni corrected post-hoc test. Due to breaches in normality we used non-parametric tests for inhibition errors during Cued tasks. A Kruskal-Wallis and further Wilcoxon test was performed to identify differences between groups and within experimental blocks. A significance level of *P* < 0.05 was established for all statistical analyses. All results and graphs are expressed as means ± standard deviations (SD). A total of 2 children did not wish to complete all the experimental blocks due to reported fatigue.

## Results

### Eye movements: EO & EH Cued vs. Non-Cued

**Fig 2:**
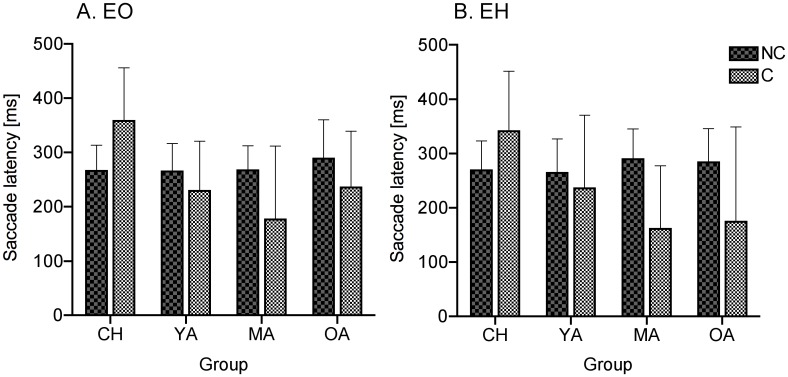
EO (A) and EH (B) Cued and Non-Cued mean saccade latencies (± SD) across the age groups. Interestingly, children’s latencies increased during Cued compared to Non-Cued tasks and are also longer compared to MA and OA, who exhibited shorter latencies during these Cued trials.

A significant interaction between age group and cued condition was apparent [*F*(3,65) = 8.682, *P* = 0.003]. Post-hoc tests showed that all groups exhibited differences in saccade latencies between C and NC tasks except for the young adults (*P* = 0.18). The analysis showed that MA and OA significantly *decreased* their saccade latencies during the C compared to the NC tasks (*P* < 0.001). In contrast, children exhibited *increased* saccade latencies during C compared to the NC tasks (*P* = 0.005) and had longer saccade latencies in this C condition compared to the other groups (*P* = 0.009, *P* < 0.001 and *P* = 0.001 for YA, MA and OA respectively) (see Fig. 2). No differences were found in eye movements between the EO and EH tasks (*P* > 0.05).

**Fig 3:**
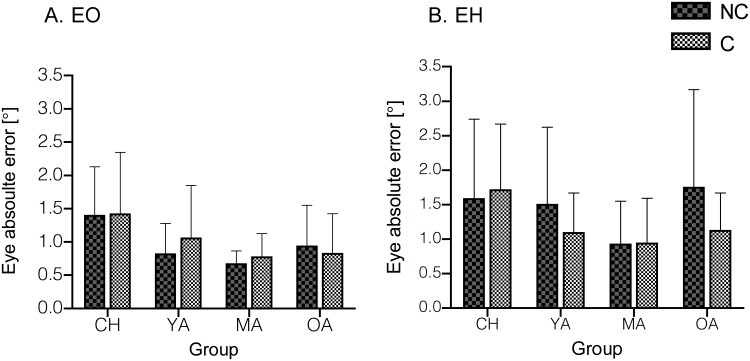
Mean eye absolute errors (± SD) during Cued vs. Non-Cued when performing the task with eyes only (A) and eyes and hand (B) across the age groups. Saccades were overall more accurate in EO compared to EH.

Saccadic absolute errors to the target showed differences between age groups when adding a touch response. The addition of the hand increased the absolute error of the eye from the target in both the NC and the C tasks [*F*(1,3) = 16.25, *P* < 0.001]. Analysis also revealed an age group effect [*F*(3,65) = 2.96, *P* = 0.039], however, the post-hoc tests only revealed significant saccade accuracy (absolute error) differences between the children and MA groups (*P* = 0.002) (Fig. 3). No task differences or significant interactions were obtained. Furthermore, saccade constant and variable errors did not reveal any differences between tasks or age groups (*P* > 0.05).

### Inhibition errors: EO, EH & HO Cued

**Fig 4:**
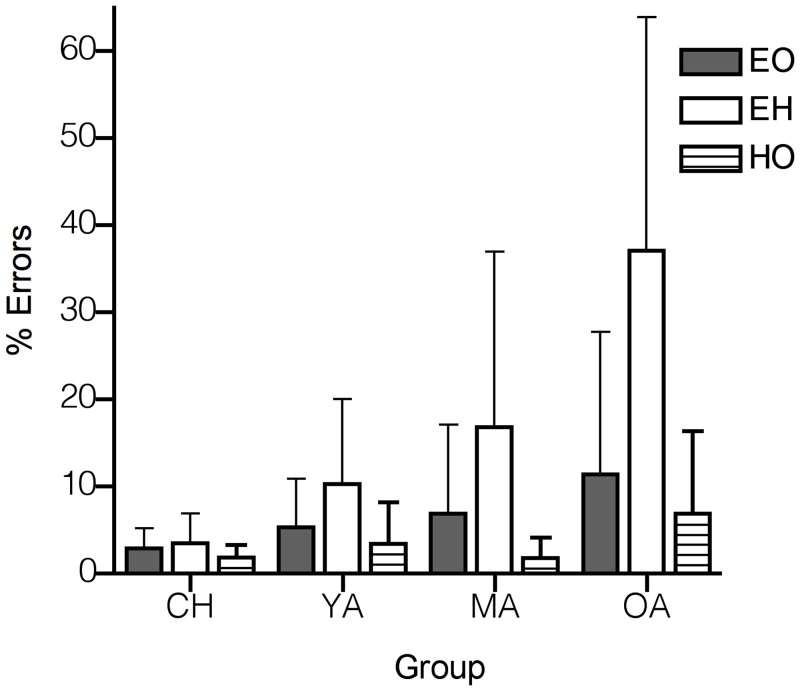
Mean inhibition errors (± SD) in response to the cue (% of trials) in EH tasks across the age groups. A linear increase in inhibition errors can be observed, particularly during coordinated actions. Older adults showed more errors during EH tasks compared to EO tasks and compared to the other age groups.

Practice effects were inspected for each age group and no significant decreases in inhibition errors were observed throughout the experimental blocks (*P* > 0.05). Fig. 4 shows the inhibition error rates across the four age groups during EO, EH and HO. We found that in EO tasks, children made less saccadic errors to the cue compared to YA (*P* = 0.024). Similar results were obtained in HO tasks, where children exhibited reduced inhibition errors compared to YA (*P* = 0.023). In EH tasks, older adults had higher error rates compared to the rest of the age groups (*P* = 0.002, *P* < 0.001 and *P* = 0.009 for YA, MA and children respectively). Furthermore, the results showed that the age groups exhibited more inhibition errors when combining eye and hand compared to EO (*P* = 0.024) and HO (*P* = 0.007).

### Hand movements: EH & HO Cued vs. Non-Cued

**Fig 5:**
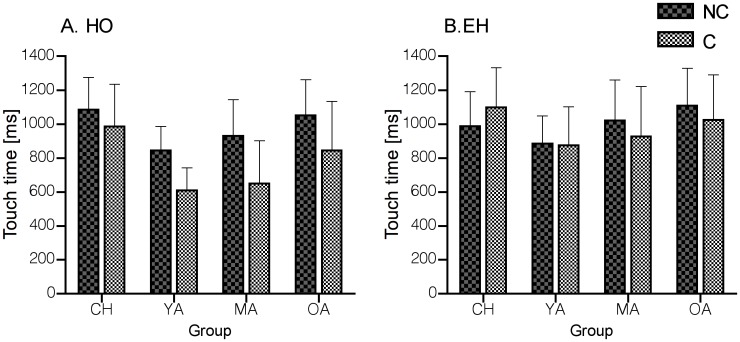
Mean Non-Cued and Cued touch times (± SD) during HO (A) and EH (B) across the age groups. Cued task effects were observed in HO (A) but not in EH. Overall, children and older adults were slower in responding to the target compared to YA.

A significant interaction between C and NC tasks and the EH and HO conditions [*F*(1,62) = 31.886, *P* < 0.001] was found. Post-hoc tests showed that touch times decreased during the C compared to the NC tasks in HO (*P* < 0.001), but touch times were not affected by the cue during EH conditions (*P* = 0.5). In addition, differences between the conditions were only observed in C tasks, with HO showing faster touch times compared to EH (*P* < 0.001) (Fig. 5). The analysis revealed that children and older adults had longer touch times compared to young adults (group effect *F*(3,62) = 6.417, *P* = 0.001). A task (C and NC) by group interaction did not reach statistical significance (*P* = 0.065).

**Fig 6:**
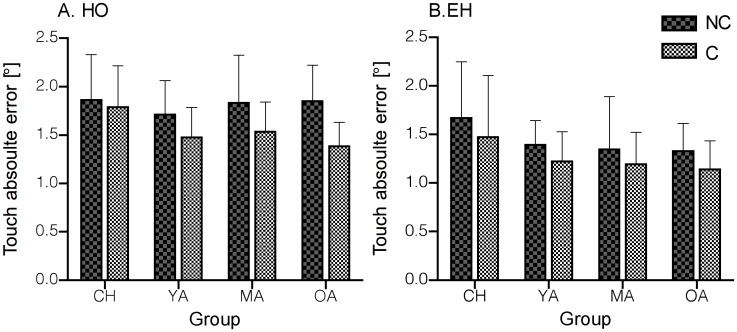
Mean Non-Cued and Cued touch absolute errors (± SD) during HO (A) and EH (B) across the age groups. The graphs illustrate better accuracy in Cued EH and HO compared to Non-Cued tasks.

Touch absolute errors in HO and EH were lower overall during the Cued compared to the Non-Cued tasks [*F*(1,62) = 24.418, *P* < 0.001]. In addition, all groups exhibited more accurate touch responses in EH compared to when the eyes were fixed, during the HO modality [*F*(1,62) = 56.33, *P* < 0.001]. A group effect [*F*(3,62) = 3.64, *P* = 0.018] revealed that children were less accurate compared to YA (*P* = 0.045) and compared to OA (*P* = 0.022) (Fig. 6). Similarly, touch constant errors [*F*(3,62) = 5.93, *P* = 0.018] revealed smaller errors in the Cued (mean-0.12 0.1) compared to the Non-Cued condition (mean 0.6 0.29) and we found that children exhibited larger errors compared to OA (*P* = 0.03) and than YA (*P* = 0.04) (means of 0.86 0.33, -0.412 0.3, and 0.292 0.3; for CH, OA, and YA respectively).

## Discussion

Our experimental paradigm revealed developmental and ageing effects on the reactive and volitional control of eye movements, touch responses and coordination of these actions. During Cued tasks, target timings and locations were predictable allowing the investigation of the developmental and ageing effects of pre-planned eye and hand movements compared to NC visually-guided responses. Results from the Cued task presented contrasting inhibitory and anticipatory effects in CH and OA, but revealed good equilibrium between these systems in YA. Our results show that the children (8–12 years) exhibited delays in the execution of a response to cued stimuli. The finding of successful inhibition of the cue agrees with previous studies that investigated the development of inhibitory mechanisms in a similar age group (~ 10 years of age) [17,18]. The delays observed in saccade initiation in children could be explained by competing brain resources between inhibiting incorrect responses and preparing a response. Age-related saccadic latency differences between adults and children have been previously reported [14,18,26,27]. Particularly, longer latencies in children versus adults have been observed in tasks that require additional cognitive control [14,17,19], in line with the Cued results presented here.

In contrast, older adults exhibited a decreased ability to inhibit saccades (as previously reported by [5,15,28]), mainly during eye and hand coordinated tasks. Hikosaka [29] found that older adults exhibited inhibition errors during the encoding phase of a memory-guided saccade task. A lowered ability to actively maintain future goals is associated with reduced frontal lobe integrity [30,31] and, in particular, goal failures have been mostly reported when attention is allocated to multiple tasks or when pre-potent behavioural tendencies are in opposition—such as the conflict in the C task between inhibition and the preparation of a coordinated motor response [32,33]. In addition to errors in inhibition, OA showed a reduced Cued saccade latency, which is consistent with the frequency of observed anticipatory responses compared to the children and younger adults, predominantly during coordinated responses (about 24% more anticipatory responses in EH). Higher inhibition error rates and shorter latencies show that the inhibition/anticipation network in the brain is not perfectly in balance in OA during saccade production [34]. Similarly, the results show that this inhibition/anticipation network is not fully developed in children.

In contrast to the CH and OA groups, young adults showed a good balance between fast reaction times with minimal errors. Movement durations for manual aiming were longer in both the children and the older adults. Longer movement times are known to be associated with aging, in particular, OAs tend to exhibit more error corrections during aiming tasks [11,35,36]. However, CH and OAs were able to program a hand response and reduce movement times in HO tasks. It is clear that OA are able to benefit from advance information and execute fast responses, however, planning a coordinated eye and hand response shows greater age-related detrimental effects.

In summary, we have used a novel eye-hand task across different age groups to establish that young children trade speeded responses for the avoidance of response errors. The cost-benefit (inhibition-anticipation) balance appears to be optimized in young adults. In contrast, older adults adopt an anticipatory strategy that produces decreased reaction times with advance information but results in a high incidence of incorrect responses. There are several studies that have investigated top-down inhibitory networks during typical anti and pro saccade tasks [4] and the inhibition of saccades has been associated with activity in the frontal and supplementary eye fields (FEF and SEF respectively) and the dorsolateral prefrontal cortex (DLPFC) [4,37–40]. These areas have also been associated with predictive mechanisms in saccadic eye movements [34,41]. There is still much research that needs to be done to understand the neural development of motor control from children to adulthood but it is likely that prefrontal cortex, medial temporal lobe and the cerebellar network will be found to be of critical importance in the control of anticipatory eye movements [41].
